# Aphasia Recovery: When, How and Who to Treat?

**DOI:** 10.1007/s11910-018-0891-x

**Published:** 2018-10-15

**Authors:** Catherine Doogan, Jade Dignam, David Copland, Alex Leff

**Affiliations:** 1 Department of Brain Repair and Rehabilitation, UCL Queen Square Institute of Neurology, London, UK; 2 Institute of Cognititive Neuroscience, UCL, 17 Queen Square, London, UK; 3 UQ Centre for Clinical Research and School of Health & Rehabilitation Sciences, The University of Queensland, St Lucia, Australia

**Keywords:** Aphasia, Neurological rehabilitation, Stroke, Speech and language therapy, Quality of life

## Abstract

**Purpose of Review:**

We now know that speech and language therapy (SALT) is effective in the rehabilitation of aphasia; however, there remains much individual variability in the response to interventions. So, what works for whom, when and how?

**Recent Findings:**

This review evaluates the current evidence for the efficacy of predominantly impairment-focused aphasia interventions with respect to optimal dose, intensity, timing and distribution or spacing of treatment. We conclude that sufficient dose of treatment is required to enable clinical gains and that e-therapies are a promising and practical way to achieve this goal. In addition, aphasia can be associated with other cognitive deficits and may lead to secondary effects such as low mood and social isolation.

**Summary:**

In order to personalise individual treatments to optimise recovery, we need to develop a greater understanding of the interactions between these factors.

## Introduction

### Aphasia

**Fig. 1:**
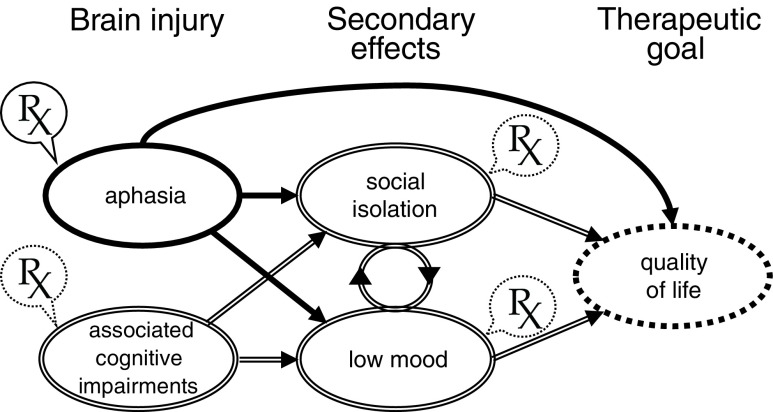
How aphasia can lead either directly or, via secondary effects, to a reduction in patients’ quality of life. Brain injury sometimes also causes other associated cognitive deficits, outside the language domain which may interact with the language impairment exacerbating secondary effects. Therapies (Rx) can be targeted at the aphasic impairment itself (solid outline) in the hope that this will also improve any secondary effects and, ultimately, quality of life. However, other forms of therapy (dotted outlines) may also be required to treat any associated cognitive impairments or the secondary effects in and of themselves

We all know what it is (an acquired disorder of language functions), but does anyone else? Chris Code and his team have been studying this for the last two decades. In a transcontinental face-to-face survey of 3483 people, they found that only 37% had heard the term ‘aphasia’ with a lowly 9% having any basic knowledge of what it is (the equivalent values for Parkinson’s disease are 96%/31% and stroke 99%/53%) [1]. Clearly more work needs to be done to raise public awareness as aphasia occurs in ~ 30% of hospitalised stroke patients [2], with an estimated 30,000 new cases of stroke-induced aphasia in the UK alone (dementia, traumatic brain injury and brain tumours are the other leading causes). It is not just the presence of aphasia that matters, the impact for the individual and their surrounding support systems can be devastating. How bad is it to have aphasia? A massive survey investigated the association between the presence or absence of 75 diseases and conditions and individuals’ quality of life scores of 66,000 long-term care residents. The highest negative relationship was with aphasia [3], ahead of cancer, Alzheimer’s disease, Huntington’s chorea and quadriplegia. Given that aphasia is a chronic condition for the majority of sufferers, it seems reasonable that precious scientific and clinical resources should be dedicated to reducing its impact, which does not stop with the patient. A community-based study of primary caregivers of stroke patients showed that those looking after aphasic patients (compared with those looking after non-aphasic patients) had significantly increased caregiver task difficulty, caregiver depressive symptoms, and, more negative stroke-related caregiver outcomes [4]. The way that aphasia affects quality of life is both multifactorial and interactive; language impairment can often lead to low mood and decreased social functioning. Interventions, therefore, can be targeted primarily at the causative impairment, with the hope that reducing this will necessarily have knock on effects [5]; or, treatment strategies can focus mainly or solely on the secondary consequences themselves (Fig. 1). In this review, we cover the current evidence for the effectiveness, dose and timing of a variety of predominantly impairment-focused interventions.

## How Do We Know Whether Speech and Language Therapy Interventions Work?

Before we review the recent evidence for this, we need to be clear about which symptoms of aphasia any given therapy is targeting, e.g. the primary effects on language and other cognition functions, or the secondary ones (mood and social interactions). When assessing studies for aphasia therapy efficacy, as well as noting which of the above element(s) are covered by the outcome measures, we think it is important to report effect sizes and not just tests of statistical significance. Standardised measures of effect sizes are scaled in terms of the variability of the study populations, whilst unstandardized or ‘simple’ effect sizes are usually reported as the raw difference in the main outcome measure. Both can be useful, the former especially when comparing across studies, but the latter is more intuitive [6]. We recommend reporting both types. A commonly reported effect size is the standardised mean differences (SMD = difference between experimental and control group means/pooled standard deviation) which is the same as Cohen’s *d* (although there are several different ways of calculating the pooled standard deviation for Cohen’s *d*, especially in repeated measures studies; see, e.g. [7]). An SMD of 1 means that the experimental group improved more than the control group by 1 standard deviation. This is considered a large effect size (generally, an effect of 0.2 is considered small, 0.5 medium and 0.8 large), but note that this will rarely if ever mean a ‘return to normal’ for the group of patients as a whole, as most are many standard deviations away from the normal range, whatever language-based outcome measure is being employed.

In a heroic piece of meta-analyses (397 pages’ worth), Brady and colleagues searched databases for randomised controlled trials pertaining to speech therapy from 1946 to 2015. Their final dataset included 57 trials comprising 3002 subjects [8••]. The headline finding is that speech and language therapy (SALT) compared with no SALT improves functional outcomes, but the SMD is small at 0.28. The largest effect size is for studies focusing on treating speech production (1.28). When SALT is compared against either a form of ‘social support and stimulation’ or different forms of SALT, benefits are much less clear. These types of comparisons are confounded by the fact that a number of interventional studies employed approaches including social support and stimulation, thereby overlapping with the ‘active control’ study arms. This has been interpreted by some to mean that either patients do not really need to be treated by trained therapists [9], or that SALT does not really work [10]. Putting aside the serious problem of low dose studies (when both arms of a study have an underpowered intervention, absence of a difference is the likeliest outcome [11]), our interpretation is based on the fact that SALT is a complex intervention. This means that it has (i) many interacting components, (ii) many practices required by those delivering or receiving the intervention and (iii) a number and variety of outcomes [12]. This makes it much harder to create a ‘placebo’ form of SALT that has all the structure of the intervention but none of its content. We currently do not know exactly why SALT works, that is, which components or combination of components drive therapy effects. To answer this, we would need a whole series of well-powered mechanistic studies; but one could question whether this approach is necessary. An analogous project would be to carefully take parts of a car away in order to find out which ones make it work; the answer is most of them do, but only in combination. That is not to say that the ‘black box’ of SALT is inscrutable; rather, we should perhaps focus on working out why patients vary in their response to a given ‘dose’ of SALT (individual variability) and then use this to drive personalising therapy.

## What About Dose, Intensity and Timing of Therapy?

The issue of dose (total amount of therapy, usually measured in contact hours) is almost always confounded with intensity (dose/time). Bhogal’s excellent meta-analysis (which has been cited > 700 times) unfortunately makes this same mistake. It is entitled, ‘Intensity of aphasia therapy, impact on recovery’ [13], yet in table 3 the authors clearly make the point that high-intensity studies (with an average 8.8 h of therapy per week, compared to 2 h for the low intensity studies) are also the high dose studies (98 vs. 44 h). Brady’s Cochrane review comes out very strongly for high versus low intensity (a staggering SMD of 11.75) but again six out of eight studies used for this intensity comparison had higher dosage and intensity versus low dosage and intensity. Of the two studies that were matched for dose, one of them compared different treatments in high and low intensity arms [14] and the other found no significant difference in the primary outcome measure for high versus low intensity treatment given in the subacute phase.

A very important dose-controlled study reported by Dignam et al. looked at intensity at 48 h of therapy (impairment, functional, computer, and group based) aphasia spread over either three (intensive) or eight (distributed) weeks. Both groups demonstrated comparable improvements on a range of measures of functional communication and communication-related quality of life; however, on a test of impairment (the Boston Naming Test) the distributed group were significantly better, both after the intervention and at 1-month follow-up [15••]. In a companion paper, the authors expound on the contrasting ‘pulls’ exerted on rehabilitative practices by the neuroscientific literature on the one hand (largely dominated by motor studies in animal models) which advocates multiple repetitions over a short period of time, and, on the other, cognitive psychology, which emphasises spaced practice [16•]. High intensity may well be required for the mass-practice, item-based approaches used to improve performance on impairment-based outcomes, but perhaps spacing these in an adaptive way, acknowledging the burgeoning evidence base for the role of sleep in language learning [17], is the way forward.

Humdrum though it may be, it is hard to see beyond dose issues when interpreting the recent trial evidence. A negative, group-randomised RCT in early post-stroke aphasic patients (*n* = 80) was assumed by the authors to reflect the early timing of therapy but could clearly have failed due to low dose (median dose = 25 h over 4 weeks) [18]. A high-profile, group-randomised, positive RCT in chronic patients (*n* = 176) found significant effects following an average of 31 h of therapy (intervention group) vs. 4.5 h (control group) over three weeks [19•]. The main outcome was a therapist-scored measure of the effectiveness of verbal communication (ANELT-A). The intervention group did significantly better than the control group (*p* = 0.0004) with a moderate standardised effect size (0.58); however, the unstandardized effect was less impressive with an average improvement of 2.6 points (+ 6.5%). The ANELT-A scale has a minimally important clinical difference value of five points. Less than half of patients made an improvement of 3 points or more on the ANELT-A. It is important that well-designed studies like this make it into high-profile journals, but we are left wondering what might have been had the intervention been of a higher dose or spread over a longer period. Similarly, a negative, group-randomised RCT study in chronic patients (*n* = 30) compared high intensity (4 h a day for 4 weeks) versus low intensity (2 h a day, also for 4 weeks) [20]. Both groups improved significantly on an impairment-based measure (with medium-to-large effects: 0.4 < Cohen’s *d* ≤ 1.4) but there was no between group difference. This result is probably best seen in light of the spaced practice debate, with higher therapy doses needing to be spread out over time to maximise their effect. This approach may also address the concern that there is increased chance of dropout or lack of compliance for higher dose treatments, particularly in early stages post-stroke, and the somewhat overlooked conclusion of the Brady et al. Cochrane review that benefits of SALT were not evident at follow-up.

How then do we determine the right dose for a particular individual and therapy at a certain stage of recovery? Rather than providing a one dose fits all approach that has resulted in the current state of hits and misses, we need to consider adoption of adaptive dose-finding or dose escalation designs, as has occurred in other fields and domains [21]. It makes intuitive sense that our trial designs should not be constrained by a set dose when we have no clear guidance as to what this should be. However, undertaking such an approach will require significant large-scale trials and being able to define when an optimal dose has been reached. In other fields this is often described in terms of adverse events or possibly fatigue, but equivalent markers will need to be determined in aphasia and may also involve identifying when a therapeutic plateau has been reached.

Regarding timing of therapy in relation to stroke onset, early rehabilitation is recommended in a number of clinical guidelines internationally. This is based on the notion of a ‘window of plasticity’ that is ‘opened’ by recent ischemia, which has been demonstrated in animal experiments in the sphere of motor recovery [22]. However, there seems to be no clear evidence for the human equivalent. In the motor domain, the negative findings of the recent very large AVERT study on very early mobilisation in stroke [23] highlight the need to be cautious when making inferences from animal recovery data to human rehabilitation. Subsequent analysis of this extensive motor recovery dataset also suggest a complex interaction between the frequency and amount of intervention at this early stage [24] that also highlights the need to avoid simply assuming ‘the earlier the better’ with respect to SALT after stroke. Recent case series [25] have confirmed what we have known for a while now, that, ‘Time post onset is not related to response to treatment for aphasia in patients’ [26]. In other words, there is no good neurobiological reason for restricting therapy to the first few months after stroke, as appears to happen in most western healthcare systems.

## E-therapies

One obvious way to up dosage and save therapists’ time is to encourage aphasic patients to use e-therapies. There are many available and they vary a lot in quality. Many, but not all, are perhaps rather narrow in scope treating only a single language input or output mode, e.g. listening, reading, speaking and writing. Despite over 100 apps or other e-therapies being available [27], we will limit our discussion here to those that have some form of peer-reviewed evidence base for the efficacy. For a recent review, see [28]. StepByStep© is a computer-based, multi-modal therapy designed for patients to use under supervision of a speech therapist. In an RCT in chronic stroke patients (*n* = 34), the intervention group practiced for 20 min, three days a week for five months (~ 25 h of spaced practice) and improved their naming ability by 20% on average [29]. Therapeutic effects were seen on trained items only. The team also carried out a cost-effectiveness assessment. The intervention cost more than usual care because of the therapist’s time required to set it up, and despite rather modest gains in quality of life units (0.14 QALYs) the therapy’s incremental cost-effectiveness ratio was favourable (~£3000 per QALY, usual cut off is £20,000 in the UK). A much larger, phase 3 trial of the same intervention has recently closed and should report results soon (Big CACTUS [30]). More recently, an off-the-shelf e-therapy for adolescents and adults with specific language impairment (phonological therapy) was paired with a pharmacological intervention (donepezil), in a double-blind, placebo-controlled, cross-over RCT. The patients (*n* = 20) were all in the chronic phase and had a clinically significant auditory perceptual deficit of language [31]. They practiced over two 5-week blocks averaging 37 h of therapy in each block. There was a small but significant generalised improvement in speech perception, with more severely affected patients benefitting more. The donepezil appeared to block the therapeutic effects of the phonological therapy. MEG scanning demonstrated that the phonological training increased synaptic gain in residual parts of the left superior temporal gyrus. One of the first ever successfully delivered aphasia e-therapies was for patients with central alexia [32], but the therapy is not currently available. A recent RCT demonstrated positive effects for a new reading app (iReadMore), based on the triangle model of reading where orthography is paired with phonology and visual semantics in a mass-practice, adaptive algorithm [33•]. Twenty-one patients in the chronic phase took part, practicing for an average of 34 h in each of two, 4-week blocks. iReadMore training resulted in an 9% improvement in reading accuracy and speed for trained words (Cohen’s *d* = 1.38) but did not generalise to untrained words. The behavioural therapy was paired with tDCS brain stimulation which showed a small, additive effect 2.6% (*d* = 0.41) for both trained and untrained words. The app will be made available for patients to use in a ‘roll-out’ trial: http://www.ucl.ac.uk/aphasialab/apps/ireadmore.html.

Thus far we have considered rather narrow e-therapies; that is, they either show item-specific effects (to be expected when mass-practice, paired-associate learning techniques are employed) or sometimes some generalisation, but even then, usually restricted to a single language modality. However, there are more holistic e-therapies available. EVA Park is a novel, pseudo-3D, virtual reality platform that contains a number of functional and fantastic locations and allows for interactive communication between multiple users [34]. Aphasic patients logon, create an avatar and meet up with their therapist in this interactive environment. Twenty patients took part in a wait-list controlled, cross-over study. Over 5 weeks, they logged an average of 41 h of engagement. The intervention produced significant gains in a functional communication measure that persisted for at least 5 weeks after the therapy finished. Effect sizes were not reported but an estimate from the two group average scores suggests an improvement of ~ 15% in functional communication. This is clearly a ‘broad’ therapy, more in keeping with what face-to-face SALTs might deliver. In this trial, students of speech and language therapy who had received 2 h of training on conversation skills delivered the therapy. Therefore, EVA Park does not solve the problem of lack of therapists’ time, but it does mean that patients and therapists can effectively interact remotely. This approach also holds promise for making therapy that is sufficiently engaging and rewarding that large amount of practice can be undertaken.

## Factors That May Explain Between-Subject Differences in Response to Therapy

Predicting the likely recovery trajectories of individual patients may be achievable in the next decade or so. Stroke has a variable effect across patients and recovery is clearly dependent on several key factors that are either intrinsic (e.g. site of lesion, cognition, including ‘capacity to learn’, mood and other personal characteristics), or extrinsic (e.g. time since stroke, therapy dose and quality) to the individual. These factors necessarily interact, but what are the most important ones? As Anna Basso remarked in an early review which focused on demographic variables and found no role for age, sex or handedness: ‘The factors that really do influence outcome are initial severity of aphasia (which is inextricably associated with the extent and the location of the lesion) and rehabilitation’ [35]. Most, but not all, aphasic stroke patients improve over time and as well as tracing these ‘natural’ recovery curves, we also wish to be able to predict the effect of therapy on these curves; that is, to identify why some patients respond better to a given therapy dose than others. A naming therapy study in 33 chronic aphasic patients used maximum possible gain on trained items as the main outcome measure (this tends to emphasise gains in more mildly affected patients) [36]. They found that a mixture of cognitive and phonological skills explained about 50% of the between-subject variance in response to therapy. Where are the missing factors? Probably in the brain itself. Aguilar and colleagues recently found that brain factors were actually more important than demographic and cognitive factors when predicting individual aphasic patients’ responses to a reading therapy, although the best predictive model included both sets of factors [37••]. Whilst not focused on treatment per se and in limited numbers, Hillis et al. [38] recently demonstrated that the influence of lesion load on aphasia recovery may be influenced by SSRIs. For patients with superior temporal and/or SLF/AF lesions, the use of SSRIs improved naming outcomes, independent of the presence of depression, initial aphasia severity, or lesion volume. Whilst preliminary, these recent findings highlight the importance of considering interactions between lesion and other factors and also suggest the need to revisit possible pharmacological approaches to boosting aphasia recovery.

In recent years, a number of functional imaging studies have sought to identify therapy-induced changes in brain activity. There is now a varied and often perplexing array of findings suggesting the role of left versus right or bilateral involvement and language versus cognitive related brain networks underlying aphasia therapy success. This diversity in findings is not surprising given that many studies employ unique therapies and imaging approaches (including functional imaging tasks and analyses) in a population of highly variable patients. Nardo et al. [39] highlighted the complexity of this issue by showing that even different therapeutic cues for word retrieval had a neural priming effect (reduced activity) in different regions within bilateral frontal networks. The notion that aphasia therapy decreases brain activity was also shown by Abel et al., [40] who found decreased activity in left-hemisphere regions that was associated with treatment induced naming improvements, suggesting increased efficiency of language-related networks. Other recent findings have highlighted the important role of interactions between domain-general cognitive networks and language specific systems in therapy-induced aphasia recovery [41]. The main challenge, which is now becoming tractable, is to investigate how demographic, cognitive, and neural factors interact to determine response to therapy on a large scale [42] and how this knowledge could be applied clinically to tailor treatments [38].

Associated cognitive difficulties and low mood are factors that can influence an individual’s engagement with the therapy. Worall found that factors that determine how well people live with their aphasia include psychosocial factors, such as social support (relation status, social network size and satisfaction) and mood (depression and anxiety) [43]. They also argue that the goal of aphasia rehabilitation should be to help the person with aphasia and their family live successfully with aphasia as well as maximising recovery of their language. This means that there are several potential targets for aphasia therapy, beyond the primary language impairment. Treating associated cognitive impairments and secondary effects of brain injury (low mood and social isolation) should also improve communication and ultimately quality of life.

## Conclusions

Whilst the effectiveness of SALT has been established, we have attempted in this review to consider why individuals’ differ in their response to a particular dose of therapy and suggest how to use this to drive personalised interventions. Consideration of individuals’ demographic, cognitive and neural profiles will assist this goal and may act as a platform to predict the effect of rehabilitation on the projectile of spontaneous recovery. Furthermore, a greater understanding of the role of dose and intensity of intervention is required to inform our practice. High-dose, massed practice may indeed be necessary to affect change for item-specific, impairment-based measures; however, the spacing and distribution of this training is also important. Sufficient dose of aphasia therapy provided over a distributed schedule may be necessary to impart long-lasting, therapeutic gains.

The approach most likely to work is a multi-faceted, holistic one that sets goals and interventions aimed to achieve these addressing all elements and their potential for interaction [44], but this council of perfection requires a lot of therapist time and more resources than most aphasic patients are currently afforded. To this end, as a community of interventionists, we need to start demonstrating clear economic benefits if we are going to convince health care commissioners to spend money on this problem; unlike ‘cancer’ and ‘dementia’ there seem to be no budgets earmarked for ‘aphasia’. This seems even more pertinent given that aphasia is a chronic condition for many people and that treatment outcomes are not related to the time post onset of stroke; therefore, interventions should be provided over an extended period, and not restricted to the first few months of recovery.
